# Blood stream infections associated with central and peripheral venous catheters

**DOI:** 10.1186/s12879-019-4505-2

**Published:** 2019-10-15

**Authors:** Jose Manuel Ruiz-Giardin, Iciar Ochoa Chamorro, Laura Velázquez Ríos, Jeronimo Jaqueti Aroca, Maria Isabel García Arata, Juan Víctor SanMartín López, Marta Guerrero Santillán

**Affiliations:** 1 Internal Medicine Department, Hospital Universitario de Fuenlabrada, Camino del Molino nº 2, 28942, Fuenlabrada, Madrid, Spain; 2 Clinical Analysis Department, Microbiology Unit, Hospital Universitario de Fuenlabrada, Madrid, Spain

**Keywords:** Bacteraemia, Central venous catheter, Peripheral venous catheter, Bloodstream infection

## Abstract

**Background:**

The purposes of this study were to determine the incidence of central and peripheral venous catheter-related bacteraemias, the relationship between the suspected and final confirmed bacteraemia origins, and the differences in microbiological, epidemiological, clinical, and analytical characteristics between the groups, including evolution to death.

**Methods:**

This was a 7-year descriptive retrospective populational study of all bloodstream infections, comparing central (CB) and peripheral (PB) venous catheter-related bacteraemias in patients older than 15 years.

**Results:**

In all, 285 catheter-related bacteraemia patients, 220 with CBs (77.19%) and 65 with PBs (22.81%), were analysed among 1866 cases with bloodstream infections. The cumulative incidence per 1000 patients-day of hospital stay was 0.36 for CB and 0.106 for PB.

In terms of the suspected origin, there was less accuracy in diagnosing catheter-related bloodstream infections (68. 2%) than those of other origins (78. 4%), *p* <  0.001. The accuracy was greater for PB (75%) than for CB (66. 2%),

Coagulase-negative staphylococci were the most frequent microorganisms in both groups but occurred 1.57 times more frequently in CB (64.1%/40.6%) (*p* = 0.004), while *Staphylococcus aureus* (23. 4%/9.5%) (*p* = 0.02) and Enterobacteriae species (15.6%/6. 3%) (*p* = 0.003) were 2.5 times more frequent in PB.

The CB patients stayed at the hospital for an average of 7.44 days longer than did the PB patients; more CB patients had active neoplasia (70. 4%/32.8%), more had surgery in the previous week (29. 2%/8. 3%), and fewer received adequate empirical treatment (53.9%/ 62.5%). Catheter was not removed in 8. 2% of CB and 3.7% of PB. On the other hand, the CB and PB patients had similar Pitt scores at blood extraction (median 0.89 versus 0.84 points, respectively; *p* = 0.8) and similar survival rates at hospital discharge (91.1% versus 90. 2%; *p* = 0.81).

**Conclusions:**

Central catheters were more frequent sources of bacteraemias than were peripheral catheters. There were important differences in the microbiological aetiology as well. PB patients received correct empirical antibiotic treatment more frequently and had a higher initial rate of correct determination of the suspected source of bacteraemia. Differences in the microbiological aetiology and empirical antibiotic treatment received, and probably catheter removal and time to catheter removal could explain why CB and PB patients had similar survival rates .

## Background

Between 15 and 30% of all nosocomial bacteraemias are associated with intravascular devices [1, 2].

It is estimated that approximately 70% of patients admitted to a hospital receive some type of venous catheter. In Spain, in the 2016 EPINE (National Study of Nosocomial Infections), 49% of nosocomial bacteraemias were related to venous catheters [3].

The prevalence of catheter-related bacteraemias is more frequent in intensive care units (ICUs) and for services such as haematology, oncology, and nephrology, as well as in university hospitals of more than 500 beds [4, 5].

There are estimates that for each central catheter used, 60 peripheral catheters are used.

Peripheral lines present complication rates ranging between 2.5% and 42%. Among these complications, up to 30% of cases comprise subcutaneous induration or phlebitis [6]. It has been observed that up to 38% of peripheral catheters may be unnecessary [7, 8].

According to various published studies, the microorganisms that most frequently cause infections related to intravascular devices are coagulase-negative staphylococci and *Staphylococcus aureus* (2/ 3 of all infections), Gram-negative bacilli (20%), and yeasts [9].

Short-duration catheters can be colonised by any of the microorganisms discussed above, while in most long-duration catheters, the prevalence of colonisation by coagulase-negative staphylococci, especially *S. epidermidis*, is above 90% [5, 9].

It was observed that the replacement strategy, including peripheral catheter replacement every 96 hours , did not provide benefits compared with the maintenance of a catheter [10].

Most published studies and clinical guidelines on catheter-related bacteraemias have focused on bacteraemias of central catheter origin, and the literature on bacteraemias of peripheral catheter origin is scarce [11, 12].

This scarcity was the reason why we decided to conduct this study.

The first objective of the study was to determine the catheter subgroup bacteraemia incidence during a 7-year period (peripheral and central catheter-related bacteraemias). The second objective was to study the relationship between the suspected origin of bloodstream infections and the final confirmed origin. The third objective was to compare the microbiology of peripheral and central catheter-related bacteraemias because the data could affect the recommendations for empirical treatment that patients with suspected bacteraemias of these origins should receive. The last objective was to compare the prognosis and differences in other epidemiological, clinical, and analytical characteristics between peripheral and central catheter-related bacteraemias, including evolution to death.

## Methods

This was a descriptive populational study of all bloodstream infections in patients older than 15 years during a period of 7 years and 4 months at a hospital located in Madrid (Spain). Hospital de Fuenlabrada is the only Hospital that attends the city of Fuenlabarada with more than 200.000 people. It means that all bacteremias are diagnosed in Hospital de Fuenlabrada. The Infectious Diseases Department gets information about all positive blood cultures once by week with the Microbiology Department and these data were recovered in a data base. After sample collection, epidemiological, microbiological (microorganism ,number of positive blood cultures and detection time of bacterial growth), clinical, laboratory, and therapeutic variables were analysed for each patient by reviewing the patient’s electronic medical records. The assessment of blood cultures was performed by a physician in the Infectious Diseases Unit.

### Definitions

-Suspected origin: The suspected source of bacteraemia was determined before the final identification of microorganisms. Suspected sources depended on the physician that initially evaluated a patient This physician prescribed blood cultures extraction and wrote his initial diagnosis. This data was obtained from the patient’s electronic records after the positive blood culture. Blood culture extraction depended on the treating physician’s criteria. The BACT-Alert detection system was used. Two pairs of bottles were drawn per patient, 20-40 mL for two pairs of bottles (4 bottles).

-Definitive source of bacteremia: The source of bacteraemia was considered clinically documented if there were focal signs and symptoms and microbiologically documented when the same microorganisms were isolated from the blood and infection site. The origins were classified as follows: intravascular (intravenous catheters), gastrointestinal, respiratory tract, renal or urinary tract, central nervous system, osteoarticular, skin and soft tissue, and gynaecological origin. When no location was confirmed or if the data were unclear, the origin was classified as unknown.

-Accuracy: the relationship between the suspected origin of bloodstream infections and the final confirmed origin. The degree of success has been calculated by dividing bacteraemias with a specific suspected source that finally had that specific source among all the bactereimias with that finally specific source. -Adequate or inadequate empirical antibiotic treatment: Adequacy was determined based on the empirical treatment administered after the isolation of blood cultures and before the arrival of an antibiogram. Treatment was classified as adequate if the infecting organism was sensitive in vitro to the ordered antibiotics at an appropriate dose and route of administration.

Pitt scale: In the assessment of the Pitt scale, the oral temperature (estimated by adding 0.5°C to the axillary temperature), the presence of hypotension or the need for vasoactive drugs, the need for mechanical ventilation, the need for cardiac resuscitation, and the mental status were evaluated. The Pitt bacteremia score is used to estimate short-term mortality in bacteremias. It is calculated at initial patient evaluation. The variables pointed range 0–14 points : temperature of 35.1–36.0°C or 39.0–39.9°C (1 point), temperature of ≤35°C or ≥40°C (2 points), mental status (alert, 0 points; disoriented, 1 point; stuporous, 2 points; comatose, 4 points), hypotension (2 points), mechanical ventilation (2 points) and cardiac arrest (4 points).

It is of interest to estimate short-term mortality ( Pitt score ) (mortality to 14 days of bacteremia) and compare it with final mortality of PB and CB (mortality produced during the treatment of bacteremia).

-Evolution to death: Mortality was considered related to bacteraemia if the death was related to bacteraemia during hospital admission.

-Diagnosis of catheter-related bacteraemia has been refered to the Clinical Guidelines of the Spanish Society of Clinical Microbiology and Infectious Diseases (SEIMC) definitions. “Catheter related blood stream infection was suspected in patients with intravenous catheters and fever, chills or other signs of sepsis, even in the absence of local signs of infection, and especially if no alternative source was identified”. Positivity of blood cultures obtained through the catheter ≥ 2 hours before those obtained from a peripheral vein with the same microorganism was highly suggestive of catheter related blood stream infection”. “In the case of skin commensals, at least 2 positive blood cultures with an identical strain are required for them to be considered a cause of bacteremia catheter related blood stream infection. In this sense patients with only one positive blood culture were considered a cause of bateremia catheter related blood stream infection if it was combined with a positive catheter hubs with the same microorganism and same antibiogram or sugestive symthoms as they have been defined before taking in account the growth time of cultures. Semiquantitative cultures of catheter hubs with ≥15 cfu (Maki method) may be indicative for catheter bacteremia if it is combined with positive blood culture with the same microorganism and same antibiogram” [13] or if the catheter was not cultured, on the presence of clinical signs of phlebitis or unexplained fever that resolved upon removal of the catheter.

The catheters could be classified as peripheral or central, with the latter including both central lines per se and peripherally inserted central lines, tunnelled or non-tunnelled. During the study period weren’t used antibiotic coated central venous catheters.

All episodes were followed until discharge from the hospital or death.

The University Hospital where the study was done is a hospital with 406 beds, and it is a category II of hospitals dependent on the Madrid Health Service, which serves an area with a population of 219,639 residents. The hospital provides inpatient beds for all medical specialties except angiology and vascular surgery, cardiovascular surgery, maxillofacial surgery, paediatric surgery, thoracic surgery, plastic and reconstructive surgery, stomatology, immunology, and neurosurgery.

### Statistics

The quantitative data were expressed as the mean and standard deviation or median and interquartile range. The Kolmogorov-Smirnov test and Shapiro-Wilk tests were used to confirm the normality of the distribution. The means were compared using Student’s t-test for quantitative variables, and the population variances were considered equal if the Levene's test value was greater than 0.05. The medians were compared using the median test.

The chi-squared test was used to describe the categorical variables. To compare proportions, the macro used was the ‘Confidence interval for the difference between two independent proportions of the Odds Ratio, 1998© J.M. Domenech’ in the SPSS® statistical program. Statistical significance was considered as a p value less than 0.05.

## Results

During the study period (7 years and 4 months), there were 101,690 hospital admissions with 609,686 hospital stays, and 1,866 bacteraemia cases were diagnosed. A total of 285 cases were catheter-related bacteraemias (15. 3%), ranking third in frequency based on the origin of bacteraemia, after urinary (560 or 30%) and digestive (352 or 18.9%) origin.

About 1854 bacteremias, 1742 (93,9%) were drawn two pairs of bottles (4 bottles) to blood cultures.

The cumulative incidence of catheter-related bacteraemias per 1,000 patients/day of hospital stay was 0.467. The cumulative incidence of central venous catheter-related bacteraemias was 0.36 per 1,000 patient days of stay, and that of peripheral catheter-related bacteraemias was 0.106 per 1,000 patient days. 1788 bacteremias had recordered information about type of catheter used, 1357(76%) had a peripheral line catheters, and 431 (24%) had central line catheters.

The relationship between the suspected origin and the final confirmed origin of the bacteraemias is shown in Table 1. There was more accuracy for bacteraemias of urinary (U) (86. 4%), gastrointestinal (G) (84. 4%), and respiratory (R) (90. 4%) origin than for those of catheter-related origin (C) (68. 2%), with the absolute difference in the percentage of certain diagnosis versus catheter-related bacteraemias as follows:

**Table 1:** Suspected and definitive source of bacteremia

		Definitive source of bacteremia										
		G	U	R	SS	UN	C	O	E	CN	GI	Total
Suspected source of bacteremia	U	17 4.8%	481* **86.** 4**%**	4 1.85%	4 4%	12 4.5%	6 2. 2%	1 5.6%	2 3.9%	0 0%	0 0%	527 28. 4%
	R	11 3.1%	19 3. 4%	198* **90.** 4**%**	2 2%	31 11.7%	26 9. 4%	0 0%	3 5.9%	0 0%	0 0%	290 15.6%
	G	297* **84.** 4**%**	14 2.5%	4 1.8%	1 1%	32 12%	15 5. 4%	1 5.6%	1 2%	0 0%	0 0%	365 19.7%
	C	3 0.9%	4 0.7%	3 1. 4%	1 1%	26 9.8%	189* **68.** 2**%**	1 5.6%	25 49%	0 0%	0 0%	252 13.6%
	SS	0 0%	1 0. 2%	1 0.5%	82* **82.8%**	9 3. 4%	2 0.7%	1 5.6%	0 0%	0 0%	0 0%	96 5. 2%
	O	0 0%	0 0%	0 0%	0 0%	2 0.8%	1 0. 4%	12* **66.7%**	1 2%	0 0%	0 0%	16 0.9%
	CNS	0 0%	0 0%	0 0%	0 0%	2 0.8%	1 0. 4%	0 0%	0 0%	11 100%	0 0%	14 0.8%
	UN	24 6.8%	37 6.6%	9 4.1%	9 9.1%	152 57.1%	37 13. 4%	2 11.1%	19 37. 3%	0 0%	0 0%	289 15.6%
	GI	0 0%	1 0. 2%	0 0%	0 0%	0 0%	0 0%	0 0%	0 0%	0 0%	4 100%	5 0. 3%
Total		352 100%	557 100%	219 100%	99 100%	266 100%	277 100%	18 100%	51 100%	11 100%	4 100%	1854 100%

*U* Urinary, *G* Gastrointestinal, *R* Respiratory, *C* Catheter, *SS* Skin and soft tissues, *O* Ostheoarticular, *CN* Central Nervous system, *E* Endocarditis, *UN* Unknown, *Gi* Ginecology

*Congruent suspected and definitive source of bacteremias are in bold text

U-C: 18.12% (95% CI, 11.94% to 24.30%), p < 0.001 in favour of the urinary focus (U). R-C: 22.18% (95% CI, 15.45% to 28.90%), p < 0.001 in favour of the respiratory focus (R); and G-C: 16.14% (95% CI, 9.47% to 22.81%), p < 0.001 in favour of the gastrointestinal focus (G).

Of the 285 catheter-related bacteraemia cases, 220 (77. 2%) were related to a central catheter (CB), and 65 (22.8%) were related to a peripheral catheter (PB).

When comparing the accuracy of determining suspected sources of bacteraemias related to central or peripheral catheters, the accuracy was greater for the PB origin (75%) than for the CB origin (66. 2%). The absolute difference in the proportions between suspicion for bacteraemias related to venous catheters was 8.8% in favour of peripheral catheters, although no statistical difference was found (95% CI, −3.56% to 21.16%) (p = 0.18).

About the number of positive blood culture bottles, patients with only one positive blood culture were 19 (8.6%) in CB group and 3 (4.7%) in PB group, without statistically significant differences p= 0.28; IC 95%( 4.6%: -2. 2% to 10. 3%). This group had a strong suspicion of catheter related bacteremias although only one bottle of cultures was positive.

Patients with two positive blood culture bottles were 57 (25.9%) in group of CB and 13 (20%) in group of PB, without statistically significant differences p= 0.33 IC 95%( 5.9%: -5. 4% to 17. 2%).

Patients with three or more positive blood culture bottles were 154 (70%) in group of CB and 49 (75. 3%) in group of PB, without statistically significant differences P 0.39 IC 95%( 5. 3%: -6.7% to 17. 4%).

The predominant microorganisms were as follows. In both groups, coagulase-negative staphylococci were the most frequent ones, at 141 (64.1%) in CB and 26 (40.6%) in PB, with a significant difference ratio of 1.57 (1.15 to 2.15). There were also significant differences in other causative agents of the bacteraemias. Enterobacteria were 2.5 times more frequent causative agents of bacteraemias associated with PB than those associated with CB (ratio 1.16 to 5.26). *Staphylococcus aureus* was 2.5 times more common for bacteraemias associated with PB than for those associated with CB (ratio 1.35 to 4.54) (Table 2).

**Table 2:** Central and peripherical catheter bacteremias and microorganisms

MICROORGANISM	CB	PB	*P % difference	% Difference (IC 95%)	P ratio	Ratios (IC95%)
Polimicrobian 21/284	17/220 (7.7%)	4/64 (6. 2%)	0.69	**(CB-PB)** 1 (− 5 to 8)	0.69	[**CB/PB]** 1.23 (0.43 to 3.54)
Enterobacterias 24/284	14/220 (6. 3%)	**10/64 (15.6%)**	**0.019**	**(PB-CB) 9.** 2**(0.** 2 **to 18.72)**	**0.02**	**[PB/CB]** 2**.5 (1.16 to 5.26)**
Staphylococcus cn 167/284	141/220 (64.1%)	**26/64 (40.6%)**	**< 0.001**	**(CB-PB) 23.46 (9.86 to 37)**	**0.004**	**[CB/PB] 1.57 (1.15 to** 2**.15)**
Enterococo 6/284	4/220 (1.8%)	2/64 (3.1%)	0.52	**(PB-CB**) -1. 3 (− 5.9 to 3. 3)	0.52	[**PB/CB]** 1.72 (0.32 to 10)
*Candida spp* 15/284	13/220 (5.9%)	2/64 (3.1%)	0.38	**(CB-PB)** 2.7 (− 2. 4 to 8)	0.39	[**CB/PB]** 1.89 (0.43 to 8.16)
*Staphylococcus aureus* 36/284	21/220 (9.5%)	**15/64 (23.** 4**%)**	**0.003**	**(PB-CB) 13.8 (**2**.8 to 24.92)**	**0.003**	**[PB/CB]** 2**.5 (1.35 to** 4**.54)**
Gram negative not fermentative 6/284	4/220 (1.81%)	2/64 (3.12%)	0.52	**(PB-CB)** -1. 3 (− 5.9 to 3. 3)	0.52	[**PB/CB]** 1.7 (0.32 to 10)

*CB* Central catheter blood stream infection

*PB* Peripherical catheter blood stream infection

IC 95% 95% Confidence interval

*Significative values are in bold text

The empirical treatment received was adequate in 53.9% of cases of CB and in 62.5% of cases of PB (p=0.25).

About catheter removal : The peripheral catheter was not removed in 2 patients (3.7%) about 53 bacteremias that had recordered this ítem. The central catheter was not removed, in 16 (8. 2%) about 195 bacteremias that had recordered this ítem. OR 2.79 IC 95% (0.5 to 10. 2) p=0.28.

When comparing Pitt scale scores of the bacteraemias related to CB (158 bacteraemias) or PB (59 bacteraemias), there were no significant differences in the Pitt scores at blood extraction (Table 3).

**Table 3:** Comparison bacteremias by central and peripheral catheter

Categorical variable (Chi squared)	Central catheter	Peripherical catheter	P *
Active neoplasia 172/277	152/216 (70. 4%)	20/61 (32.8%)	**< 0.001**
Steroids 45/259	41/199 (20.6%)	4/60 (6.7%)	**0.01**
Immunosuppressants 56/263	52/203 (25.6%)	4/60 (6.7%)	**0.002**
Diagnostic speciality bacteremia			**< 0.001**
- Medical (93/281)	52/218 (23.9%)	41/63 (65.1%)	**< 0.001**
- Surgical (80/281)	68/218 (31. 2%)	12/63 (19%)	0.059
- ICU (53/281)	50/218 (22.9%)	3/63 (4.8%)	**0.001**
- Hematology –Oncology (36/281)	31/218 (14. 2%)	5/63 (7.9%)	0.18
Quantitative variables (Median and interquartile range 25-75%). U-Man Whitney			
Days with the last catheter	**12 (6.25–42.25)**	4 **(**3**–7)**	**< 0.001**
Days in Hospital to bacteremia	**14 (**6-**30)**	**7 (**4**–13)**	**0.02**
Days from bacteremia to adecuate antibiotic treatment	1 (0–3)	0 (0–2.5)	0.16
Hospital admissions > 48 h in the last 12 months (not including actual admission)	1 (0–2)	0 (0–1. 2)	**0.014**
Pitt Score	0 (0–1)	0 (0–1)	0.54
Time to positivity (hours)	13.92 (10.56–18)	13.92 (10.8–17.04)	0.99
Urea (mg/dL)	42 (31–55.7)	38 (27–58)	0.27
C- reactive protein (mg/dL)	10.87 (4.9–21. 4)	8.10 (3.12–14.57)	0.082
Leukocytes (*10*^*3*^ /μL)	7500 (3630–12,400)	7800 (5950–12,650)	0.52
Platelets (*10*^*6*^/μL)	177 (75–343)	169 (134.5–255.5)	0.91
Quantitative variables (Student t test)	Mean difference Central and Peripherical catheter		
Age (years)	1.8 (−2.58 to 6.18)		0.41
Systolic blood pressure (mmHg)	-5. 2 (− 12.58 to 2.08)		0.16
Dyastolic blood pressure (mmHg)	−1.71 (− 5.65 to 2.22)		0.39
Creatinin (mg/dL)	−0.08 (− 0.34 to 0. 17)		0.51
Hemoglobin (g/dL)	**−0.78 (− 1.45 to − 0.1)**		**0.02**

*Significative values are in bold text

At hospital discharge, survival was recorded for 275 patients (96.5% of all catheter-related bacteraemia patients). In all, 91,1% of the patients with CB and 90. 2% of those with PB were alive (p= 0.8). There weren’t statistically significant differences in Pitt score either final mortality in peripehrical and central catheter related bacteremias.

When we compared the mortality between the PB and CB patients, depending on empirical antibiotic treatment (it was recorded for 245 (85.9%) patients, related CB 189 (85.9%), and related PB 56 (84,6%) patients), the risk to die was 2,75-fold (95% CI, 0.42-18) higher if a PB patient received inadequate antibiotic treatment than if the patient received adequate antibiotic treatment (p = 0.25). In this group, the mortality increased from 5.7% to 14. 2% with inadequate empirical antibiotic treatment.

The risk to die was 1.32-fold (95% CI, 0.48-3.59) higher if a CB patient received inadequate antibiotic treatment (88 of 189 patients with 9 deaths) than if the patient received adequate antibiotic treatment (101 of 189 patients with 8 deaths) (p = 0.58). In this group, the mortality increased from 8% to 10. 2% with inadequate empirical antibiotic treatment. If a PB patient received inadequate antibiotic treatment (21 of 66 patients) the mortality increased from 5. 3% to 14. 2% ( p=0.27).

### Other variables

The median number of days of dwell time related to CB was 12 (IQR 6.25–42.25), and that related to PB was 4 (IQR 3–7) (p < 0.001).

Regarding the inpatient units where the bacteraemias occurred, the majority of infections related to PB occurred in medical inpatient units compared with bacteraemias related to CB, which predominantly occurred in intensive care units (ICUs), surgical units, and haematological units (Table 3).

The patients with a CB-related bacteraemia had a mean stay of 7.44 days (95% CI, 1.09–13.79) longer than that of the patients with a bacteraemia related to PB.

Also significant was the number of hospital admissions for more than 48 hours in the last 12 months, with more admissions in the last 12 months for patients who are diagnosed with a bacteraemia related to CB than for patients with a PB-related bacteraemia.

Comorbidities were present in 93.5% of the patients with CB and included active neoplasia in 70. 4%. In the PB patients, comorbidities were present in 85. 2% (active neoplasia in 32.8%) (p< 0.001).

No analytical differences were noticed in blood tests (haemoglobin, leucocytes, platelets, renal function, and C-reactive protein levels) or haemodynamic parameters (systolic and diastolic blood pressure) during bacteraemia (Table 3).

## Discussion

Catheter-related bacteraemias involve increased hospital stays, costs, morbidity, and mortality [14].

Catheter-related bacteraemias are one of the most frequent cases of nosocomial bacteraemias and are classically associated with the use of central venous catheters, mainly in intensive care units.

In recent years, the incidence of catheter-related bacteraemias in other hospital units, as well as their relationship to the use of peripheral venous catheters, has also increased [15].

It has been estimated in different studies that the use of peripheral venous catheters is 60 times more common than that of central catheters; however, the rates of bacteraemia related to peripheral catheters are lower than those of bacteraemia related to central catheters. In our study, central catheters were responsible for 77% of catheter-related bacteraemias compared with 23% for peripheral catheters. These data are similar to those of the study on the prevalence of nosocomial infections in Spain (EPINE, 2016), in which data recovered from 294 hospitals and 59,016 patients showed that among all catheter-related bacteraemias (306 bacteraemias), 73.85% (226 bacteraemias) were related to central venous catheters and 26.14% (80 bacteraemias) were related to peripheral catheters [3]. In our study, the cumulative incidence of catheter-related bloodstream infections per 1,000 patients/day of hospital stay was more than 3 times higher for central catheters than that for peripheral catheters. This finding concurs with the data reported in other studies, such as that by Pujol et al [16], whose estimated rate was 0.18 episodes of bacteraemia per 1,000 days of peripheral catheter use at a university hospital; a 5 times higher rate, 0.9 episodes per 1,000 days of central catheter use, was recorded simultaneously in another study [16].

This difference may occur because the initial appearance of peripheral phlebitis is usually a physicochemical phenomenon [17, 18]. This initial peripheral phlebitis is associated with a low risk of initial infection, although it generally entails a change in the venous access route, decreasing the dwell times of peripheral venous catheters, which could lead to a lower incidence of bacteraemias associated with peripheral catheters. In our study, there were significantly higher catheter dwell times for central catheters compared with those for peripheral catheters (a median of 7. 4 more days). In the study by Targer et al., an increased risk of phlebitis occurred between the second and the third day of catheter insertion and remained stable thereafter, which is the reason why these authors recommend changing peripheral lines every 48 to 72 hours [19]. However, subsequently, the data from randomised controlled prospective studies have been published in which systematic replacement every 72 hours was compared with that performed when clinically indicated. There were no differences in terms of the number of cases of phlebitis, dysfunctional catheters, and local infection or bacteraemia rates [20, 21].

Regarding clinician's diagnostic accuracy, clinicians have a lower diagnostic accuracy of suspected sources when bacteraemias are due to venous catheters than if bloodstream infections are due to other sources of bacteraemias.

Our study showed a clinician's diagnostic accuracy of over 85% when the bacteraemia source was respiratory, urinary, or gastrointestinal and a lower accuracy when the bacteraemia source was intravascular (congruencies of approximately 68%). This may explain why only 53.9% of the CB patients and 62.5% of the PB patients received appropriate empirical antimicrobial treatment against microorganisms associated with venous catheter-related bacteraemias. There were more accuracy for PB origin than for CB origin probably because of macroscopic manifestations of flebitis are more frequently associated to peripheral venus catheter bacteremias than with central ones.With respect to the microorganisms associated with catheter-related bacteraemias, coagulase-negative staphylococci, followed by *Staphylococcus aureus*, were the most frequent aetiological agents. *Staphylococcus aureus* was 2.5 times more common for bacteraemias associated with PB than for those associated with CB. This is congruent with another published studies. Akihiro Sato et al. [22] described 62 peripheral venous catheter related bloodstream infeccions. Gram positive microorganism were responsable of 58% (*S aureus* 17%) peripheral venous catheter related bacteremias (in our study *S aureus* is responsable of 23% of PB). In different studies a mean of 38% (range, 12–64%) of *S. aureus* catheter related bacteremias were related to peripheral venous catheter and a mean of 19% (7.6–35%) of *S. aureus* bacteremias were due to infected peripheral venous catheter [23].

About gram negatives PBs, Ripa el al [24] described that gram negative microrganisms were responsible of 22.8% of peripheral venous catheter related bloodstream infections. In our study is next to 19%. And in the study of Tsuboi M et al [25] gram negative rods were more frequently identified in peripheral venous catheter related bloodstream infections than in central ones (33%-18.8%) . In this study peripheral catheters were regularly replaced at least once every 96 hours and it could explain a relative higher amount of gram negative related bacteremias in front of longer peripheral catheter duration. In our study gram negative bloodstream infection are also more frequent in peripheral venous catheter related infections than in central ones ( 18.7% and 6. 3%). In our study 50% of PBs were related to more than 96 hours placed peripheral venous catheter, and it could explain a lowest relative gram negative bloodstream infections in advance of gram positives. There are studies that relates peripheral catheter dwell time and risk of *S. aureus* bacteremia. A study found a significant higher risk of *S aureus* bacteremias related to peripheral venous catheter if the median dwell time was over 3 days .[26]. In this sense a study of 137 *S. aureus* peripheral catheter related bacteremias noted that 45% involved peripheral venous catheter in situ beyond 4 days and that 61% had been inserted by the ambulance service or in the emergency department [27].

The reason for this association is not clearly defined, although it may be related to the modification of the cutaneous microflora as the days of hospitalisation are extended as well as to simultaneous antibiotic therapy that the patient receives [26]. In this sense, it is also striking that 15% of PBs and 8% of CBs were caused by enterobacteria, which should be taken into account when planning empirical antibiotic treatment.

In terms of the inpatient units where bacteraemias occurred, those associated with peripheral catheters mainly occurred in internal medicine inpatient units, while bacteraemias associated with central venous catheters occurred in those specialised units where interventions predominate or prolonged parenteral treatment is required, as is the case for ICUs, oncology, or haematology. In this respect, we observed that active neoplasia was present in 70. 4% of the patients with central venous catheter-related bacteraemias.

In general, patients with catheter-related bacteraemias have higher central catheter dwell times and prolonged hospital stays compared with patients with bacteraemias associated with a peripheral catheter.

The clinical status of the patients who suffer from a bloodstream infection associated with a catheter is similar for PB and CB, as we showed in our study, in which both groups had similar Pitt scores (0.89 in CB and 0.84 in PB) without differences in arterial blood pressure or creatinine, urea, and haematological values at the time of bacteraemia.

As we mentioned before, 53.9% of the CB patients and 62.5% of the PB patients received appropriate empirical antimicrobial treatment. The percentage of adequate empirical treatments was lower for CB than for PB, a nearly 10% difference with no statistical significance, probably due to the lower number of peripheral venous catheter-related bacteraemia cases. These cases, nevertheless, demonstrated survival rates close to 90% (CB, 91.1% and PB, 90. 2%).

Adequate empirical antibiotic treatment is a well-defined factor in bacteraemic survival. Therefore, the question is how it is possible that patients with different proportions of adequate empirical antibiotic treatment (CB, 53.9% and PB, 62.5%) have similar survival rates.

And in this sense in our study CB have more Hospital stay, active neoplasia, esteroids and others inmunosupressant treatments than PB. CB have also longer time to receive specific adequate treatment than PB. And CB are more frequently diagnosed in Intensive Care Units, Surgical departments, Haematology and Oncology deparments . But although all these ideas could make us to think about a worse prognosis in CB, they have the same prognosis than PB. The probable answer is that survival rates are influenced by several factors. The first is that at the time of bacteraemia, both groups had similar Pitt scores, without differences in blood test parameters such as creatinine, urea, or haematological values. The second factor is that microorganisms, such as coagulase-negative staphylococci, were more frequent in CB, at 141 (64.1%), than in PB, at 26 (40.6%). This factor could explain a better prognosis for the CB patients, even though they received fewer adequate empirical antibiotic treatments. In this sense there is a tendency toward higher mortality among patients with PB and who did not received adequate empiric therapy . It may be in part explained by the higher frequency of *S. aureus* in PB. It is described that PB due to *S. aureus* are significantly more likely to have a metastatic focus of infection , and greater mortality [16] and a significantly duration bacteremia compared to cases of non-PVC-related *S. aureus* bacteremia [23, 28].

And the third factor is catheter removal and the proportion of early catheter removal.that is probably one of the most important factors for outcome of catheter-related bloodstream infection.

About this point it would be interesting to know the number of days since the bacteremia to catheter removal but this item was not recordered.

Establishing a therapeutic recommendation thus requires further studies as well as preventive measures to reduce the incidence of bacteraemia associated with this type of device, which is so frequently used in hospitalised patients [15, 23].

About anothers limitations of the study , the first one is the retrospective nature of the study.

During the study period there were 101,690 184 hospital admissions with 185 609,686 hospital stays. It would be of interest knowing how many patients had central lines in this period, and not only in the bacteremic patients. Usually in the Hospital the most part of patients use peripheral lines and we only use central ones if long periods of time are needed for antibiotic tratment, chemotherapy, parenteral nutrition, or incapacity to use peripheral venous lines.

We found that the mortality was higher in patients who received inadequate empirical antibiotic, particularly in PB patients. It would be of interest knowing if all the patients were treated adequately thereafter (antibiotics adjusted according to susceptibility of the organism), how long were they treated if there was any persistent bacteremia or any resistant organism. Also it would have been of interest to know how was the catheter managed not only if it was retired or not . Finally as we have mentioned before it would be of interest knowing the number of days since the bacteremia to catheter removal because early catheter removal is probably one of the most important factors for outcome of catheter-related bloodstream infection.The study group with 285 catheter-related bacteraemia patients may seem relatively small and highly unbalanced, with 65 PB and 220 CB bacteraemia patients; however, this is a populational study, which means that variables with clinical and statistical differences are very strong from a statistical point of view. The proportion is also congruent with that in the EPINE 2016 study [3].

## Conclusions

Peripheral catheters can be sources of catheter-related bacteraemias.

Central catheter-related bacteraemias are more frequent than peripherical catheter-related bacteraemias, although peripherical catheter devices are used more frequently than central catheter devices.

Catheter-related infections are the third most frequent source of bacteraemias in our hospital, with high error margins in initial determination of the suspected source and in empirical antibiotic treatment prescriptions.

There was more accuracy in diagnosing bacteraemias of urinary, gastrointestinal, and respiratory origin than those of catheter-related origin, and there was more accuracy for PB origin than for CB origin.

Enterobacteria and *S. aureus* were more frequent causative agents of bacteraemias associated with a peripheral venous catheter. Coagulase-negative staphylococci were more frequent among the causative agents of bacteraemias associated with central catheters.

PB patients received correct empirical antibiotic treatment more frequently and had a higher initial rate of correct determination of the suspected source of bacteraemia. Differences in the microbiological aetiology and empirical antibiotic treatment received, and probably catheter removal and time to catheter removal could explain why CB and PB patients had similar survival rates .

## Data Availability

The database supporting the current study are available at the Hospital Universitario de Fuenlabrada. Madrid. Spain. The datasets used and analysed during the current study available from the corresponding author on reasonable request.

## Abbreviations

C: Catheter origin
CB: Central venous catheter related bacteraemias
G: Gastrointestinal origin
PB: Peripheral venous catheter-related bacteraemias
R: Respiratory origin
U: Urinary origin
